# Differentiation syndrome-induced Myopericarditis in the induction therapy of acute Promyelocytic leukemia: a case report

**DOI:** 10.1186/s40959-021-00124-9

**Published:** 2021-11-23

**Authors:** Shabari Mangalore Shenoy, Thomas Di Vitantonio, Anna Plitt, Rocio Perez-Johnston, Jillian Gutierrez, David A. Knorr, Eytan M. Stein, Jennifer E. Liu, Stephanie Feldman

**Affiliations:** 1 Department of Medicine, Mount Sinai Morningside and West Hospital, Icahn School of Medicine at Mount Sinai, New York, USA; 2 Department of Medicine, New York Presbyterian/ Weill Cornell Medical Center, New York, USA; 3 Division of Cardiology, Department of Medicine, Memorial Sloan Kettering Cancer Center, New York, NY USA; 4 Department of Radiology, Memorial Sloan Kettering Cancer Center, New York, NY USA; 5 Leukemia service, Department of Medicine, Memorial Sloan Kettering Cancer Center, New York, NY USA; 6 Division of Cardiology, Department of Medicine, Memorial Sloan Kettering Cancer Center. Weill Cornell Medical College, New York, NY USA

**Keywords:** Myopericarditis, Differentiation syndrome, All trans retinoid acid, Arsenic trioxide, Acute Promyelocytic leukemia

## Abstract

**Background:**

All trans retinoic acid (ATRA) has revolutionized the treatment and outcomes of patients with Acute Promyelocytic Leukemia (APL). Induction therapy with ATRA is associated with the rare but potentially fatal complication of differentiation syndrome. While the presentation of this syndrome is varied, myopericarditis as a manifestation of differentiation syndrome is often fatal and rarely reported in literature. We present a case of myopericarditis as the sole manifestation of differentiation syndrome in a patient on induction therapy with ATRA and arsenic trioxide for APL.

**Clinical presentation:**

A 62 year old woman with remote history of breast and uterine cancer presented to the hospital for expedited work up of easy bruising and expanding hematomas. She was diagnosed with APL with peripheral blood and bone marrow cytogenetics revealing t (15;17) translocation and initiated on induction therapy with ATRA and ATO as well as steroids for differentiation syndrome prophylaxis. Eighteen days into induction therapy, patient developed pleuritic chest pain, elevated cardiac biomarkers, ECG changes suggestive of pericarditis. Cardiac magnetic resonance imaging showed patchy multifocal sub-epicardial late gadolinium enhancement and elevated T2 signal consistent with acute myopericarditis. Given the timing of symptom onset and lack of other identifiable cause, patient was diagnosed with differentiation syndrome- induced myopericarditis and promptly initiated on high dose steroids with rapid improvement in symptoms, ECG, and cardiac biomarkers. Patient successfully resumed dose-reduced ATRA and arsenic trioxide without complication.

**Conclusion:**

Myopericarditis can be the sole manifestation of differentiation syndrome and the presentation may be atypical owing to the use of prophylactic steroids as illustrated in our patient’s case. A high index of suspicion for differentiation syndrome, multimodality imaging, and prompt input from multidisciplinary providers is crucial for making the timely diagnosis and initiating life-saving treatment.

## Introduction

Differentiation syndrome (DS) is a potentially life-threatening complication of induction therapy with all-trans retinoic acid (ATRA) for acute promyelocytic leukemia (APL) [1]. Common cardiac complications of the combination of ATRA and arsenic trioxide (ATO) include DS-related pericardial effusions and ATO-mediated QT prolongation [2]. Myopericarditis secondary to differentiation syndrome is rarely reported in the literature [3–7]. We present an unusual case of myopericarditis as the solitary manifestation of DS in a patient receiving ATRA and ATO for APL.

## Case

A 62-year-old woman with a history of breast cancer treated with modified radical mastectomy, chemo-radiotherapy (doxorubicin, cyclophosphamide and paclitaxel) and tamoxifen about seventeen years ago and uterine carcinosarcoma treated with total abdominal hysterectomy with bilateral salpingo-oophorectomy and no other pre-existing cardiovascular disease or risk factors presented to our hospital for expedited workup of 1 week of easy bruising and expanding hematomas. Upon presentation she was tachycardic and hypertensive with physical exam notable for scattered ecchymoses of various sizes. Initial laboratory evaluation revealed pancytopenia with hemoglobin of 10.2 g/dl, platelet count of 12,000/L, white blood cell (WBC) count of 1.5 × 10 9/L, and absolute neutrophil count of 0.7 × 10–3/ml. Fibrinogen and INR were 50 mg/dl and 1.9, respectively. A presumptive diagnosis of acute promyelocytic leukemia (APL) was suspected based on the presence of increased promyelocytes on bone marrow smear. She was started on all-trans-retinoic acid and her coagulopathy was stabilized with blood products. The diagnosis of APL was confirmed with peripheral blood and bone marrow cytogenetics revealing a t (15;17) translocation.

**Fig. 1:**
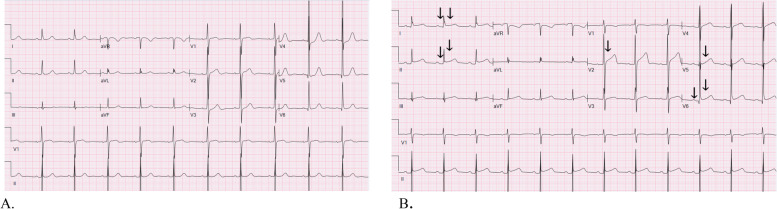
**A** Baseline (pre-treatment) ECG: normal sinus rhythm, left ventricular hypertrophy by voltage criteria, QTc 433 ms. **B** ECG on day + 18 of ATRA/ATO regimen: normal sinus rhythm, left ventricular hypertrophy, QTc 448 ms, diffuse ST segment elevation, PR depression (arrows)

**Fig. 2:**
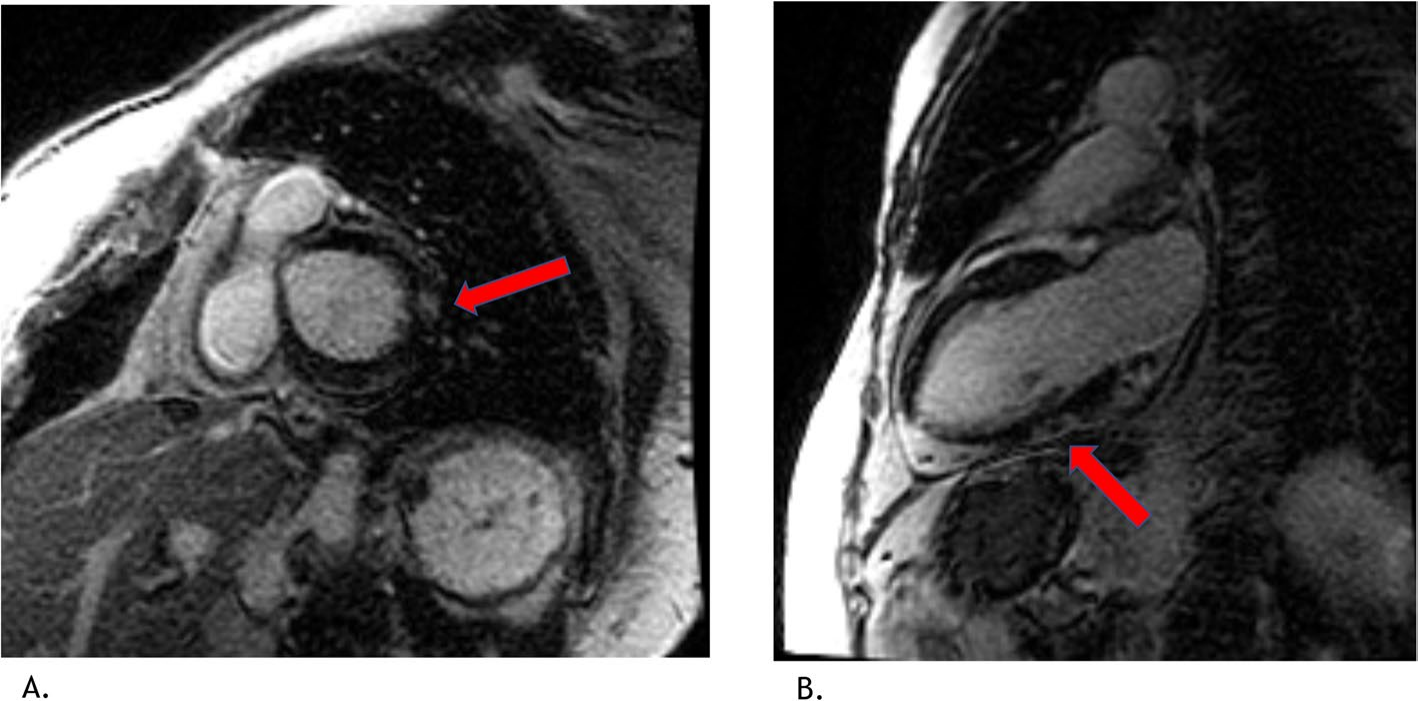
**A** Short axis view, basal left ventricle, sub epicardial late gadolinium enhancement (LGE) in the basal anterolateral wall (arrow). **B** 2 chamber view, sub epicardial LGE in the mid inferior wall (arrow)

She was classified as low/intermediate risk (WBC < 10 × 10 9/L) and started on ATRA (45 mg/m2/day) and ATO (0.15 mg/kg) for 5 days per week, with differentiation syndrome prophylactic prednisone (0.5 mg/kg daily) [2, 8]. In the early phase of induction therapy, she had intermittent episodes of atypical chest pain and shortness of breath. During these episodes serial troponins were negative and electrocardiograms did not demonstrate ischemic changes. However, on Day 18 of induction therapy, the quality of her symptoms changed, now described as chest pain at rest, waking her from sleep and radiating to the neck lasting several minutes. The pain was worse when supine and relieved by sitting upright. Vital signs and physical exam were within normal limits, cardiovascular exam without murmurs, rubs or signs of volume overload. Electrocardiogram demonstrated sinus tachycardia, diffuse ST segment elevations and PR segment depressions (Fig. 1) while laboratory studies revealed troponin I elevated to 11 ng/ml (normal range < 0.02) and leucocytosis to 26.3 × 10 9/L. A transthoracic echocardiogram demonstrated preserved biventricular function, no regional wall motion abnormalities, and minimal pericardial effusion. Her clinical presentation was deemed most consistent with acute myopericarditis given the pleuritic nature of her chest pain, elevated troponin, ECG changes suggestive of acute pericarditis, normal wall motion and minimal pericardial effusion on the echocardiogram. To further evaluate for etiology of myopericarditis, an infectious workup was performed with blood and urine cultures, respiratory viral panel including SARS COVID-2, chest radiograph, all of which returned negative. Given the timing of her symptom onset and the negative work-up to identify another etiology, DS-induced myopericarditis was highly suspected. She remained on prophylactic prednisone through the development of symptoms. Due to the potentially life-threatening nature of DS, ATRA and ATO were held and she was immediately started on high dose steroids (dexamethasone IV 10 mg every 12 h) for the treatment of DS and colchicine 0.6 mg daily for the treatment of pericarditis. To confirm the diagnosis of acute myopericarditis, the patient underwent a cardiac magnetic resonance imaging (MRI) which demonstrated normal biventricular function with no regional wall motion abnormalities, trace pericardial effusion, patchy multifocal sub-epicardial late gadolinium enhancement of the basal anterolateral and mid inferior walls of the left ventricle and elevated T2 signal suggestive of inflammation (Fig. 2). These findings on the cardiac MRI were consistent with the diagnosis of acute myopericarditis. Our patient’s symptoms resolved within 2 days of high dose dexamethasone initiation, the troponin level returned to undetectable levels after 10 days and the WBC count returned to normal range. With troponin, WBC count, ECG, echocardiogram within normal limits, treatment with ATRA and ATO was resumed at a two-dose level reduction using ATO 0.10 mg/kg and ATRA 25 mg/m2 (per the Lo Coco protocol) without complication [2]. Dexamethasone was changed to prednisone 60 mg daily and was the patient was gradually weaned off of it with tapering the dose by 10 mg every week.

## Discussion

To our knowledge, this is the first case of myopericarditis from differentiation syndrome in a patient treated with ATRA and ATO reported in the literature. This case highlights the importance of maintaining a high index of clinical suspicion of DS in patients with APL being treated with ATRA and ATO. It is crucial to be cognizant of the time of onset and various presentations of this syndrome especially in patients on prophylactic steroids due to their anti-inflammatory effect against cytokines released from differentiating myeloid cells leading to a subtle but potentially life-threatening presentation [9].

Although the use of ATRA and ATO induction therapy has revolutionized the treatment of APL, a once essentially fatal malignancy, and led to complete remission along with improvement in disease-free and overall survival; differentiation syndrome (DS) is one of the major adverse effects of ATRA therapy and/ or arsenic trioxide therapy [10]. DS is caused by ATRA-induced differentiation of promyelocytes to more mature myeloid forms accompanied by massive cytokine release and a systemic inflammatory response. This potentially fatal syndrome was first documented and described by Frankel et al. in 1992 [9]. The incidence of DS was as high as 26% in patients treated with ATRA monotherapy during the induction phase and symptoms appeared anywhere between 2 and 21 days from starting chemotherapy [9].

Differentiation syndrome frequently poses a diagnostic dilemma for clinicians due to the absence of specific diagnostic criteria and its varying manifestations. It is suggested that DS should be considered in patients with APL on ATRA and/or ATO in the presence of two or more of the following signs or symptoms: fever, weight gain, respiratory distress, pulmonary infiltrates, pleural or pericardial effusion, hypotension, or renal failure [11].

Dyspnoea, fever, pulmonary infiltrates are found in greater than 50% of the reported cases. Our patient developed pleuritic chest pain without other typical signs or symptoms of differentiation syndrome such as fever, hypotension, weight gain, respiratory distress, pulmonary infiltrates and pleural effusions which is potentially attributable to the use of prophylactic steroids [12]. Her presentation was unique in that all prior reports of DS related myopericarditis have been in patients treated with induction regimens consisting of cytotoxic chemotherapy along with ATRA, while she was treated with ATRA and ATO [3–7]. There are also a limited number of reported cases of isolated myopericarditis without concomitant pericardial effusions [5–7].

Given the atypical presentation of differentiation syndrome, a multidisciplinary team that included oncologists, cardiologists, pharmacists, cardiac imagers, and multimodality imaging with echocardiogram and cardiac MRI were key to making a timely diagnosis and early initiation of DS directed treatment. It was also important to rule out other potential etiologies for DS such as infection prior to interrupting cancer directed treatment and initiating high dose steroids. Rapid resolution of our patient’s symptoms with initiation of dexamethasone, as well as normalization of ECG and troponin provided further support for the diagnosis of DS-induced myopericarditis.

## Conclusion

Myopericarditis is a rare manifestation of differentiation syndrome in a patient with APL receiving treatment with ATRA and ATO. Myopericarditis should be included within the spectrum of DS as rapid recognition and treatment is crucial to improving patient outcomes. Furthermore, patients on steroid prophylaxis may present with atypical presentations, and a high index of suspicion must be maintained to allow for early recognition and initiation of life-saving therapy. This rare manifestation of a potentially lethal condition highlights the crucial role of a multidisciplinary care team and the use of multimodality imaging to make the diagnosis.

## Data Availability

Not Applicable.

## Abbreviations

ATRA: All Trans Retinoic Acid
APL: Acute Promyelocytic Leukemia
ATO: Arsenic Trioxide
DS: Differentiation syndrome
ECG: Electrocardiogram
MRI: Magnetic resonance imaging
WBC: White blood cell
